# Daytime and public space exposure to *Anopheles funestus* bites in Western Province, Zambia: implications for malaria surveillance and control

**DOI:** 10.1186/s12936-025-05363-0

**Published:** 2025-04-18

**Authors:** Benjamin Chanda, Keith J. Mbata, Javan Chanda, Busiku Hamainza, Megan Littrell, Joseph Wagman

**Affiliations:** 1 PATH, Kaoma, Zambia; 2 School of Natural and Applied Sciences, Department of Biosciences and Biotechnology, University of Zambia, Lusaka, Zambia; 3 PATH, Lusaka, Zambia; 4 National Malaria Elimination Centre, Lusaka, Zambia; 5 PATH, Washington, DC USA

**Keywords:** Persistent malaria transmission, Residual malaria transmission, *Anopheles funestus*, Western Zambia

## Abstract

**Background:**

Western Province, Zambia experiences persistent and residual malaria transmission despite high household coverage of core vector control interventions. Standard vector surveillance, conducted overnight at households, indicates that the dominant malaria vector *Anopheles funestus *sensu stricto (*s.s*.) bites opportunistically both indoors and outdoors, and remains active throughout the night and into the late morning after the sun rises. This suggests that the full extent of community exposure to *An. funestus s.s.* bites may not be well characterized in Zambia. This study piloted an expanded vector surveillance approach to capture 24 -hour biting patterns at households and in public spaces, including schools and markets, where core interventions offer limited protection.

**Methods:**

Monthly mosquito collections were made in two rural villages and two peri-urban neighborhood-based clusters in Western Province, Zambia, from January to April 2024. Paired indoor-outdoor human landing catches were implemented over 24-hour periods. A total of 9600 collection hours were performed at randomly selected households, with 768 additional collection hours performed equally across two school and two market buildings.

**Results:**

A total of 2305 female *Anopheles* mosquitoes from 11 morphologically differentiated species were collected, with *An. funestus *sensu lato (*s.l*.) the most abundant (41%, 942). Aggregated across all hours of the day and all locations there was an overall average of 6.9 *An. funestus s.l.* bites/day (b/d) (95% CI 5.0–8.8), with comparable rates indoors (3.52 b/d) and outdoors (3.37 b/d). Similar rates occurred at home (2.27 b/d), school (2.38 b/d), and market (2.25 b/d). While 87.6% of bites (6.0 b/d) occurred overnight (1800–0600 h), 12.1% (0.83 b/d) occurred during daylight hours between 0600–1100 h.

**Conclusion:**

Results document significant exposure to *An. funestus* bites both indoors and outdoors, at home as well as in public spaces such as schools and markets, and late in the morning until 11:00 h in Western Province, Zambia. The flexible blood-feeding behaviours exhibited by this dominant malaria vector highlight important operational gaps in the protection offered by current vector control strategies that are deployed primarily indoors and/or during nighttime hours. Vector surveillance efforts should be extended to better characterize the full scope of transmission risk throughout the community and guide the development of new approaches to target transmission occurring outdoors, during the daytime, and in public spaces away from the home.

**Supplementary Information:**

The online version contains supplementary material available at 10.1186/s12936-025-05363-0.

## Background

Malaria remains a critical public health issue, particularly in sub-Saharan Africa where it contributes significantly to morbidity and mortality [1]. The World Health Organization (WHO) recommended malaria vector control interventions, namely insecticide-treated nets (ITNs) and indoor residual spraying (IRS), contributed significantly to the reduction of malaria transmission and disease burden observed between 2000 and 2015, together averting over 500 million clinical cases of malaria in that period [2]. Despite this considerable achievement, progress towards further disease reduction targets has stalled and the continued impact of ITNs and IRS is compromised in many communities by residual and persistent transmission [3], which is defined as the continued transmission of malaria following the implementation of a widely effective control programme [3]. Several factors are thought to contribute to residual and persistent transmission, including insecticide resistance as well as various vector (such as daytime and outdoor biting) and human (such as variable sleeping patterns and bed net use) behaviours that can limit the effectiveness of these core interventions [4, 5]. Furthermore, mosquito behaviours are dynamic and known to change in response to efforts undertaken to control them, which can exacerbate gaps in protection and further complicate vector control operations [6].

Addressing current operational gaps in protection necessitates development of complementary malaria vector control tools and approaches. Achieving sustained control and eventual elimination requires a multifaceted strategy that enhances infrastructure, improves environmental management, and strengthens health systems. Additionally, engaging communities and promoting health education on malaria prevention are crucial [7]. A comprehensive and integrated vector control effort is essential to effectively tackle the complexities of malaria transmission and ensure long-term success. Foundational to these efforts is a solid understanding of local malaria transmission dynamics, including a comprehensive characterization of vector bionomics and human exposure to potentially infectious mosquito bites.

Underscoring many of these challenges are findings from the recent phase III cluster-randomized controlled trial (cRCT) of attractive targeted sugar bait (ATSB) in Western Province Zambia [5, 8], which indicate persistently high rates of malaria transmission despite high household coverage with ITNs and IRS. Indeed, in the study area malaria case incidence was greater than two per year per child in children under 5 years old and community infection prevalence remained greater than 50%, even though 97% of households either owned at least one ITN or had received IRS in the prior 12 months and over 70% of the population reported regularly sleeping under an ITN [5]. These results are in line with data collected during the most recent (2021) Zambia Malaria Indicator Survey [9], which estimated that across Western Province rapid diagnostic test (RDT) based malaria infection prevalence remained between 40% and 63% even though 70.9% of households owned at least one ITN and 55.3% of all household members indicated having slept under an ITN the previous night.

In the communities of Western Province, *Anopheles funestus *sensu stricto (*s.s.*) is the dominant malaria vector, responsible for more than 95% of all infectious bites [8, 10]. *Anopheles arabiensis* and *Anopheles gambiae s.s.* are also present, as are potential vectors *Anopheles squamosus* and *Anopheles coustani* [8, 10]. Robust entomological surveillance during the ATSB trial, which followed standard practice of collecting mosquitoes overnight for 12 h in and around participating households [11], estimated an overall entomological inoculation rate (EIR) of 2.75 (95% CI 2.12–15.2) infectious bites per household per month for *An. funestus*. Importantly, *An. funestus* was shown to opportunistically feed both indoors and outdoors, biting consistently throughout the night and into the early morning hours even when collections stopped at 06:00 h [8]. These flexible feeding behaviours are of note considering recent work in southern Central African Republic, western Kenya, Malawi, and Tanzania which has demonstrated significant biting rates for several malaria vectors, including *An. funestus,* during daytime hours and also in public and outdoor spaces away from the home [12–15].

The use of human landing catch (HLC) for extended hours (beyond the standard 12 h, overnight collections) and in public spaces outside of the home (particularly at schools) has been invaluable in helping to define a more complete picture of community-wide malaria transmission risk [8–10]. In Zambia, these broader aspects of local vector bionomics have yet to be defined, as malaria vector surveillance activities to date have been performed only at nighttime, in and around households, as is standard practice [11].

To leverage the findings and lessons learned during the ATSB cRCT, the present pilot study was designed to capture data, for the first time in Zambia, on the 24 -hour biting behaviours of *Anopheles* spp. mosquitoes, both indoors and outdoors as well as at households and in public spaces, such as schools and markets, where people are not adequately protected by current ITN and IRS-centered vector control strategies. This comprehensive surveillance approach was designed to help define key characteristics of persistent and residual malaria transmission and illustrate the limitations of current malaria vector control strategies, highlighting existing gaps in protection. Findings also aim to inform the development and adoption of expanded vector surveillance and control strategies by the National Malaria Elimination Programme and guide the development of novel vector control methods and new products.

## Methods

### Study site

**Fig. 1:**
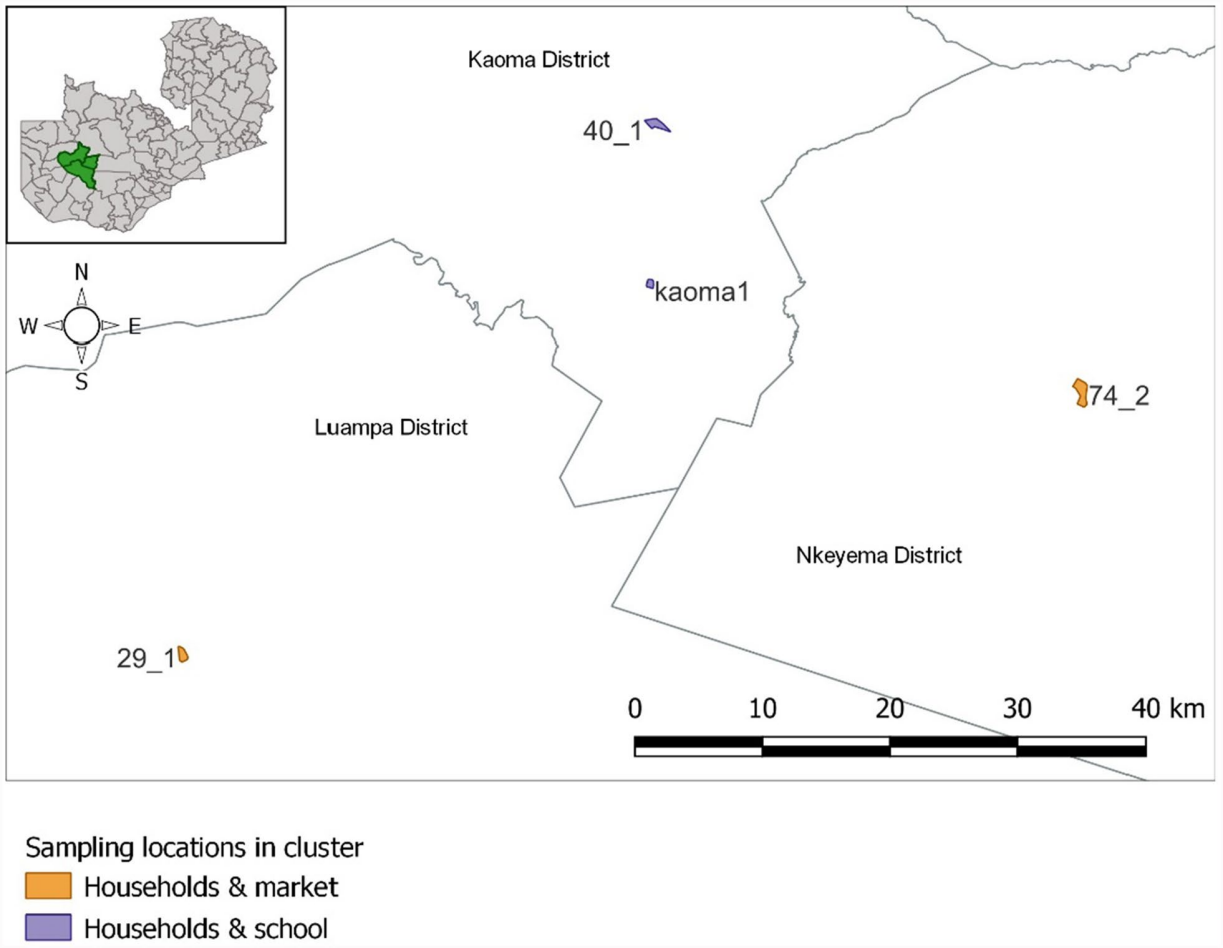
Map of the study area in Zambia

The study was conducted in two rural villages and two peri-urban neighbourhood-based clusters in Western Province, Zambia located in Kaoma, Luampa and Nkeyema districts (Fig. 1). These four communities were well characterized during the recently completed ATSB cRCT study, and are among Zambia’s highest burden provinces, with estimates of under-5-year-old rapid diagnostic test (RDT)-based malaria infection prevalence between 40% and 63% [9]. The region has a tropical savannah climate with a hot-wet season (November to April), a cold dry season (May to July), and a hot-dry season (August to October) [10]. Malaria transmission in the study site is typically high from December to May of each year, with a peak in malaria transmission from April to May [9] corresponding to the annual rainy season. The average annual rainfall is 1000 mm. The average temperature ranges from 10 to 38 °C. The main vegetation type found in these districts is miombo woodland [16].

### Cluster selection

Four clusters (Fig. 1; cluster 29_1 in Luampa District, cluster 74_2 in Nkeyema District, and clusters Koama 1 and 40_1 in Kaoma District) were selected based on specific criteria: (i) ensuring at least one cluster from within each of the three study districts, and (ii) each cluster had to include either a school or a public market. Additionally, clusters were chosen based on their proximity to the central laboratory located at the Kaoma PATH Sub-Office to simplify 24-h travel and supply logistics.

### Entomological surveillance and sampling effort

This study employed repeated cross-sectional entomological survey, collecting data over four months from January to April 2024. Mosquitoes were collected using paired indoor-outdoor human land catch (HLC) procedures over 24-h periods by trained field workers, ensuring consistent methodology and minimizing observer bias.

This study was embedded within a larger surveillance effort focused on generating robust estimates of mosquito densities in and near household sleeping structures. As such, there was an imbalanced sampling effort which utilized 9600 total collection hours at households supplemented with 768 total collection hours in public spaces, described in more detail below.

### Household level collections

A total of 25 different households (HH) were sampled per cluster per month, with five different randomly selected HH surveyed on each of the five alternating 24-h collection days. This approach resulted in a total of 9600 HH collection hours. At each HH, paired indoor and outdoor HLC collections were performed for 24 h starting at 18:00 h and ending at 18:00 h the following evening. Household identification and permission were obtained from the heads of households previously enumerated during the ATSB cRCT, at least one day before the start of each collection period.

**Fig. 2:**
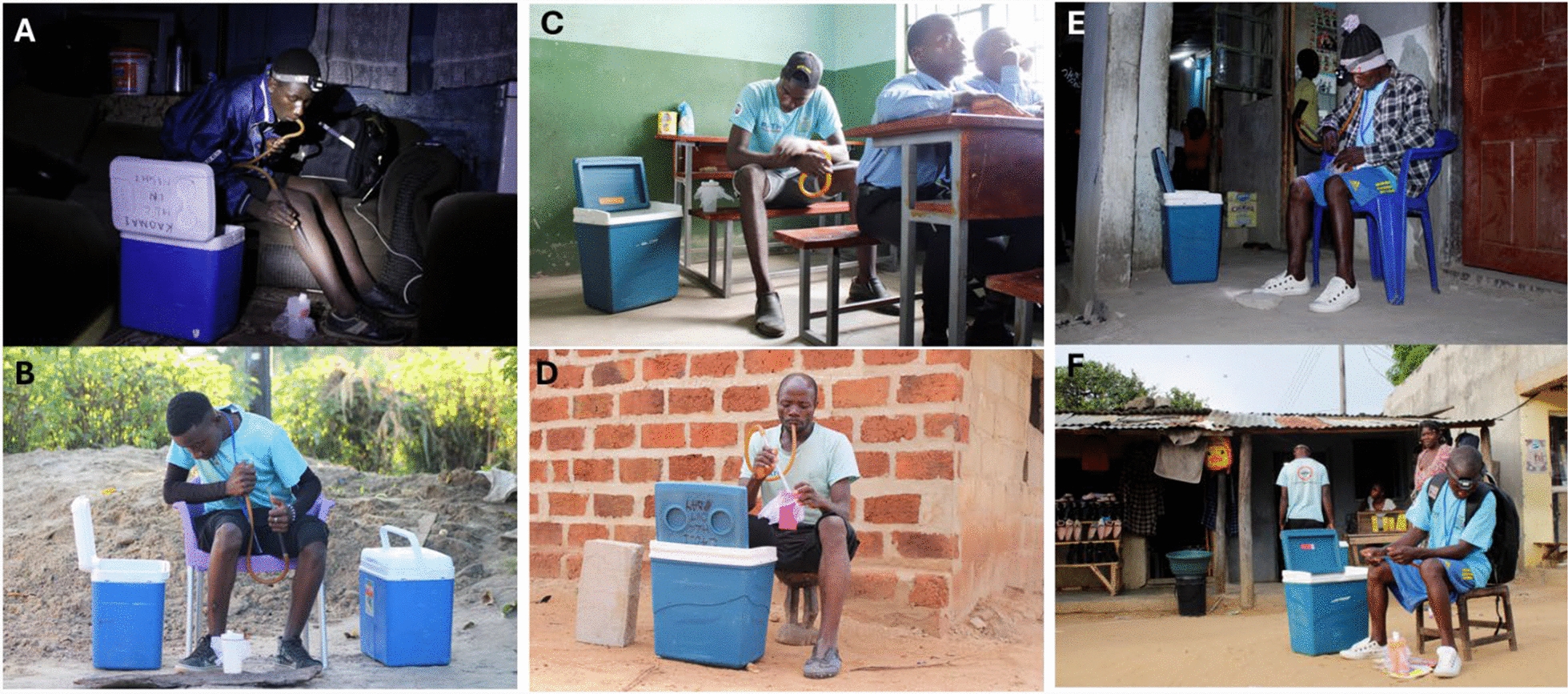
24-h human landing catch (HLC) activities occurred indoors and outdoors at households (A and B), schools (C and D), and in public marketplaces (E and F). Photo credit: PATH/Mundia H. Masuzyo

Entomology collectors worked in two shifts. Shift one run from 18:00 h to 06:00 h and shift two was from 06:00 h to 18:00 h. During both shifts, indoor collectors were situated inside a HH sleeping structure, in a room adjacent to the HH entrance, while outdoor collectors were situated outside of the occupied HH structure, in the peri domestic space with a minimum distance of 5 m from the entrance (Fig. 2a, b).

Mosquitoes were collected for 45 min of every hour, and the remaining 15 min served as a break allowing collectors to rest and enter data into study specific CommCare (Dimagi Inc., Cambridge, MA) data collection tools loaded onto Android Tablets, and prepare materials for the next hour’s collection.

Indoor and outdoor mosquito collectors sat quietly with a torch for scanning landing mosquitoes on their legs every minute and a mouth aspirator to collect mosquitoes. Collected mosquitoes were transferred to an appropriately labelled collection cup for each hour and location. For each HLC shift, all collection cups were then placed into a cluster-specific labelled, humidified cooler box for temporary storage and final transportation to the central field laboratory for further processing (Fig. 2a, b).

### Public space collections

In one public area of each cluster, at school buildings in clusters 40_1 and Kaoma_1 and at public market buildings in clusters 29_1 and 74_2 (Fig. 2c, f), paired indoor-outdoor HLC collections were implemented in two shifts over 24 h following the same procedures used at household level (Fig. 2c, f). Mosquitoes were collected over one 24-h day each study month at each location, resulting in a total of 768 public-space collection hours. Collections were coordinated in advance with local authorities, including school and market administrators, community leadership, health facility staff, community health workers, and ministry of education personnel with the authorization from the District Education Board Secretary’s office (DEBS).

### Ethical considerations for human land catch entomology collectors

Before the commencement of mosquito surveillance activities, each entomology collector completed study-specific training and provided written consent to participate in the study. In each cluster, one or two days before each month’s first collection night, all entomology collectors were tested for malaria using a histidine rich protein-2 antigen detection rapid diagnostic test (RDT; Bioline™ Malaria Ag Pf, Abbott Diagnostics). Collectors that tested negative for malaria were assigned to participate in that month’s collection and given one standard tablet of 12.5 mg pyrimethamine BP/100 mg dapsone BP Deltaprim™, Zimbabwe Pharmaceuticals Ltd) as malaria chemoprevention. Those who tested positive for malaria were excluded from participating in that month’s collection and were given a standard course of 80 mg artemether/480 mg lumefantrine (LonArt®, Bliss GV Pharma Ltd).

### Mosquito processing

At the end of each HLC collection shift, labeled collection cups were placed in appropriately labeled cooler boxes humidified with a damp towel to enhance survival during transportation to the central field laboratory in Kaoma town. Mosquitoes were rapidly knocked down via mechanical shaking of the collection cup. All field collected anopheline and culicine mosquitoes were then differentiated morphologically and enumerated.

Individual *Anopheles* mosquitoes were further morphologically identified to species using the appropriate dichotomous key [17]. For each anopheline mosquito collected the time, date, and location was recorded. Each anopheline was also classified based on abdominal appearance as unfed, partly fed, fed, or gravid [11] and stored on silica gel in individual 1.7 ml Eppendorf microcentrifuge tubes (Sigma co. Ltd) for future analysis.

### Data quality assurance, storage, and analysis

Nightly and daily spot checks were conducted by the cluster-based entomology supervisor and entomology staff to help ensure high quality data and to provide rapid troubleshooting during mosquito collection. Digital data entry utilized Android tablet devices and study specific CommCare data collection tools. Automated CommCare-created Excel dashboards were used for weekly data review and quality checks. CommCare datasets were exported to Microsoft Excel (Microsoft Corporation, Redmond, WA) and cleaned, transformed, and summarized by descriptive statistics using Excel and Tableau Desktop 2024.2 (Tableau Software LLC, Mountain View, CA), and analyzed using StataSE 15 (StataCorp LLC, College Station, TX). As appropriate, summary statistics are presented with 95% confidence intervals estimated with robust standard errors accounting for clustering at the study cluster level. Biting rates are estimated using the average number of adult female vectors that attempted to feed per person per hour stratified by location, and hourly rates are aggregated to 24- h daily rates and to nighttime and daytime when appropriate. Biting rate ratios were estimated using a simple generalized linear Poisson model with a Log link, including cluster-level random effects [18].

## Results

### *Anopheles* species diversity and relative abundance

A total of 2305 *Anopheles* mosquitoes from 11 morphologically differentiated species were captured during the study (Table 1). *Anopheles funestus s.l.* was the most abundant, accounting for 41% (942) of all specimens. *Anopheles squamosus* was the second most abundant species at 21%, (485), followed by *Anopheles tchekedii* (20.7%, 478) and *Anopheles tenebrosus* (8%, 185). *Anopheles gambiae s.l.*, which in Western Province includes *An. gambiae s.s.* and *Anopheles arabiensis*, accounted for 7.0% of all specimens (156), while *Anopheles coustani* accounted for 2.0% (46). Additional species collected in low numbers included *Anopheles gibbinsi*, *Anopheles brunipes*, *Anopheles maculipalpis*, *Anopheles flavicosta*, and *Anopheles pharoensis*, each of which accounted for less than 1% of the total collected.

**Table 1:**
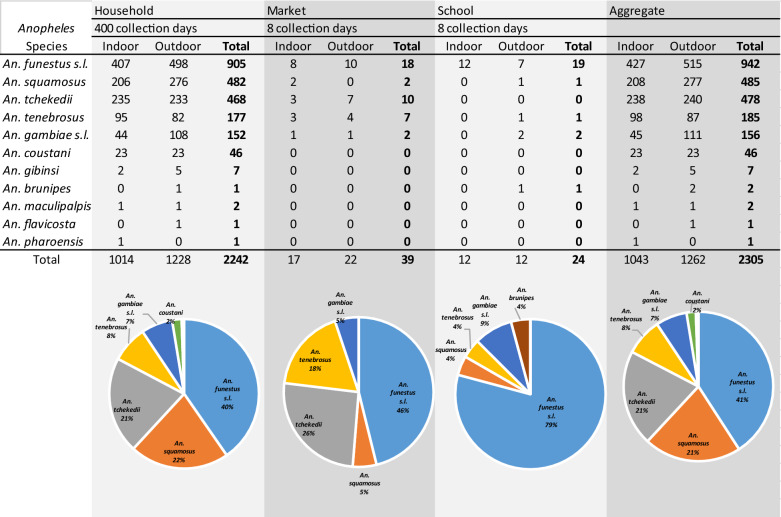
Species composition and abundance of *Anopheles* collected during the 24-h human landing catch collections

The diversity of human-biting *Anopheles* spp. was greatest at households, where all 11 morphologically identified species were captured. Fewer species were collected at public markets (5 of 11 species collected) and school buildings (5 of 11 species collected), but any differences in species diversity by location should be interpreted with caution given the reduced sampling effort in public locations. Of note, however, is that the dominant vector species *An. funestus s.l.*was the most abundant species encountered at each location, both indoors and outdoors. *Anopheles squamosus* and *An. gambiae s.l.* were also encountered at each location, while *An. coustani* was only collected at households.

### Daily biting rates of malaria vectors in Western Province

**Fig. 3:**
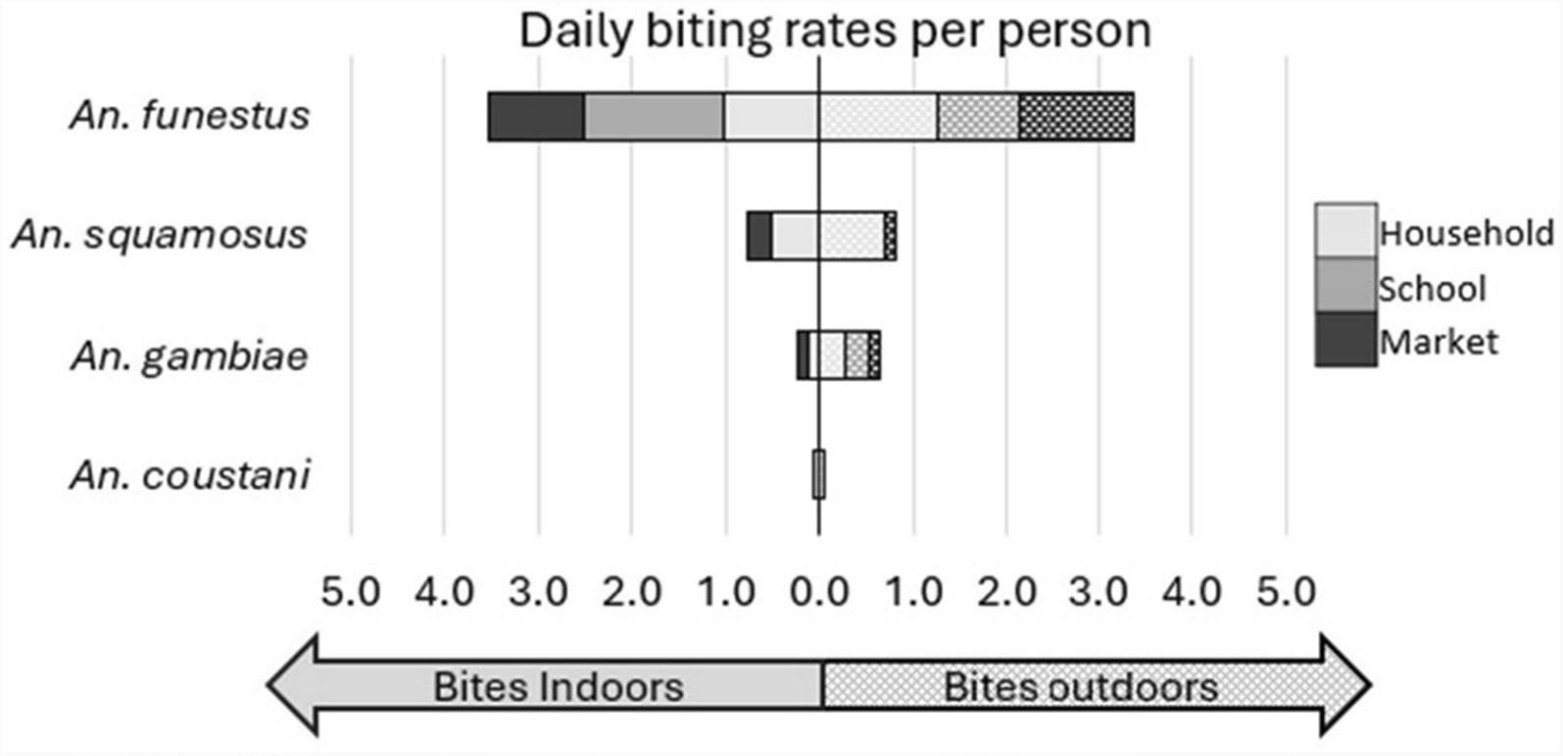
Daily biting rates, per human landing catch collector, for the locally relevant malaria vectors in Western Province, Zambi

The average number of bites per 24-h day (b/d), estimated from the data in Table 1, are shown for each species collected in Additional file 1: Table S1 and for malaria vectors in Table 2 and Fig. 3. Biting intensity was highest for *An. funestus* s.l., with an aggregate of 6.90 b/d: 2.27 at home (33%), 2.38 at school (34%), and 2.25 at market (33%). The indoor-to-outdoor biting rate ratio (RR) for *An. funestus* s.l. was not significantly different from 1.0 at home (0.821; 95% CI 0.289–2.33, *p* = 0.717) or in the marketplace (0.837, 95% CI 0.245–2.86, *p* = 0.777), though at school more bites were recorded inside the classroom compared to outdoors (RR = 1.75; 95% CI 1.69–1.80, *p* ≤ 0.001). There were 0.88 aggregate *An. gambiae* s.l. b/d, 0.38 at home and 0.25 each at school and at market, also with bites more likely to occur outdoors at home (RR = 0.410; 95% CI 0.232–0.720, *p* = 0.00).

**Table 2:** Daily bites per person recorded for the locally relevant malaria vectors in Western Province, Zambia

		Total bites	Total HLC person-days	Bites per person-day			Indoor:outdoor rate ratio^1^	95% CI	*p*-value^2^
				Total	Indoors	Outdoors			
*An. funestus* s.l	Household	905	400	2.27	1.02	1.25	0.821	(0.289–2.33)	0.717
	School	18	8	2.38	1.50	0.88	1.740	(1.69–1.80)	< 0.001
	Market	19	8	2.25	1.00	1.25	0.837	(0.245–2.86)	0.777
	**Aggregate**	**n/a**	**n/a**	**6.90**	**3.52**	**3.38**	**0.834**	**(0.312**–**2.23)**	**0.717**
*An. gambiae* s.l	Household	152	400	0.38	0.11	0.27	0.41	(0.241–0.698)	0.00
	School	2	8	0.25	0.00	0.25	–	–	–
	Market	2	8	0.26	0.13	0.13	–	–	–
	**Aggregate**	**n/a**	**n/a**	**0.89**	**0.24**	**0.65**	**0.41**	**(0.232**–**0.720)**	**0.00**
*An. squamosus*	Household	482	400	1.21	0.52	0.69	0.751	(0.710–0.795)	< 0.001
	School	2	8	0.00	0.00	0.00	–	–	–
	Market	1	8	0.38	0.25	0.13	–	–	–
	**Aggregate**	**n/a**	**n/a**	**1.59**	**0.77**	**0.82**	**0.757**	**(0.721**–**0.795)**	**< 0.001**
*An. coustani*	Household	46	400	0.12	0.06	0.06	1.01	(0.706–1.44)	0.968
	School	0	8	0.00	0.00	0.00	–	–	–
	Market	0	8	0.00	0.00	0.00	–	–	–
	**Aggregate**	**n/a**	**n/a**	**0.12**	**0.06**	**0.06**	**1.01**	**(0.705**–**1.44)**	**0.964**

^1^Indoor to outdoor landing rate ratio estimated from the generalized linear model

^2^Probability that the true rate ratio is not 1.0

– = Analysis not performed because of low sample numbers

Among other potential malaria vectors, biting rates were highest for *An. squamosus*, with an aggregate 1.58 b/d: 1.21 at home, 0.25 at market, and 0.13 at school, with bites more likely occurring outdoors than indoors at home (RR = 0.757; 95% CI 0.721–0.795, *p* ≤ 0.001). Biting rates for *An. coustani* were low, with 0.12 b/d, all recorded at home and equally likely indoors or outdoors (RR = 1.01; 95% CI 0.706–1.44, p = 0.964).

### Hourly biting trends of *An. funestus s.l.* in Western Province

**Fig. 4:**
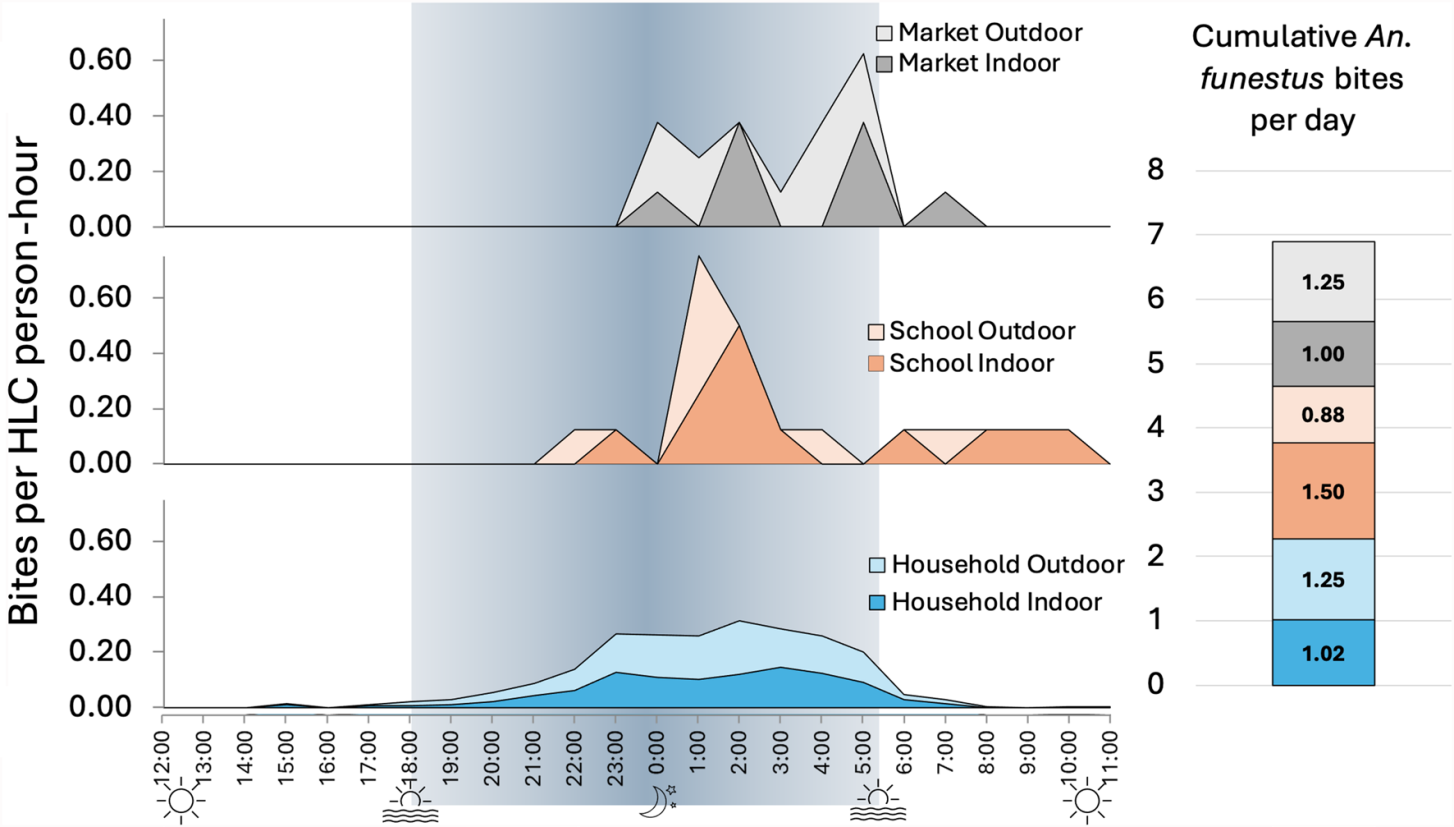
Hourly (left) and cumulative daily (right) *An. funestus* s.l*.* bites per person as recorded during 24-h human landing catch collections at home (bottom), school (middle), and market buildings (top) in Western Province, Zambia

Analysis of hourly biting patterns, illustrated in Figs. 4 and 5, shows that *An. funestus s.l.* began biting people at home, both indoors and outdoors, between 15:00 h and 16:00 h. Rates increased and remained consistently highest overnight from 22:00 h to 06:00 h, though lower biting rates were observed regularly until 11:00 h.

At school, *An. funestus s.l.* began biting during the nighttime between 22:00 h and 23:00 h. Peak biting occurred between 00:00 h and 04:00 h both indoors and outdoors, though regular biting continued at lower rates until 08:00 h outdoors and until 11:00 h indoors.

At the market, *An. funestus s.l.* began biting during the nighttime between midnight and 01:00 h, both indoors and outdoors. Peak biting was observed outdoors between 04:00 h and 06:00 h, and biting activity ceased outdoors between 05:00 h and 06:00 h and indoors between 07:00 h and 08:00 h.

Looking at the proportional breakdown of daily *An. funestus s.l.* bites recorded by hour and location (Table 2; Additional file 1: Fig. S1) highlights roughly equivalent biting exposure indoors (51% of bites [95% CI 28–74%], or 3.52 b/d [95% CI 2.2–4.8]) and outdoors (49% [95% CI 26%—72%], or 3.37 b/d [95% CI 1.9–4.8]). Biting rates were also roughly equivalent at home (2.27 b/d [95% CI 1.9–2.6 b/d]), at school (2.38 b/d [95% CI 1.0–3.7 b/d]), and at market (2.25 b/d [95% CI 0.92–3.6 b/d]). While the majority of bites (87.6% [95% CI 61.3–100%], equivalent to 6.0 bites per day [95% CI 4.2–7.8 b/d]) occurred overnight between 18:00 and 06:00 h, a significant proportion (12.1% [95% CI 3.4–20.1%]) occurred in the late morning between 06:00 and 11:00 h (equivalent to 0.83 b/d [95% CI 0.24–1.4 b/d]). Very few bites were recorded between 11:00 and 17:00 h (0.3% [95% CI 0.1–0.5%], equivalent to 0.02 b/d [95% CI 0.01–0.04 b/d]).

## Discussion

This is the first study to present findings from surveillance of 24-h human biting patterns of malaria vectors in Zambia, providing critical insights into local transmission dynamics associated with *An. funestus*, the dominant vector in Western Province, as well as several other potential vectors. By extending the conventional surveillance timeframe to include daytime hours and by including diverse settings such as schools and markets, results highlight several key aspects of vector bionomics that can contribute to residual and persistent malaria transmission in Western Zambia: there is clear risk of exposure to vector bites outdoors, in the daytime hours (particularly late in the morning between 06:00 h and 11:00 h), and in public spaces away from the home and the ITN and IRS-centered malaria vector control interventions deployed there. These findings support similar observations recently reported in Central African Republic [12], western Kenya [13], Malawi [14], and Tanzania [15], and taken together have significant implications for updating malaria surveillance and control strategies in a variety of transmission settings across Africa, including in Zambia.

The study identified 11 morphologically distinct *Anopheles* species, with *An. funestus s.l*., the dominant malaria vector [8, 10], being the most abundant across households, schools, and markets, and at all times of the day, underscoring the need for a variety of interventions tailored to its diverse behavioural and ecological traits. Its abundance in all sampled environments highlights its ability to exploit a wide range of habitats and human activities. For instance, the nearly equal distribution of *An. funestus s.l.* bites per person per day across households (2.26), schools (2.38), and markets (2.25) underscores its versatility, presenting a significant challenge for vector control programmes that focus on specific settings. This is because *An. funestus s.l.* has consistently been reported as the dominant and major vector in the same study areas of Western Zambia, transmitting about 95% of all infectious bites, with an estimated overall entomological inoculation rate (EIR) of 2.75 (95% CI 2.12–15.2) infectious bites per household per month [8, 10].

The notable presence of secondary vectors such as *An. squamosus,* and *An. coustani*, whose role in residual transmission has been reported by others [19–23] further highlights the complexity of malaria transmission in the region. For example, *An. squamosus* demonstrated an outdoor preference with a biting rate of 1.58 bites per day, predominantly at households. This behaviour suggests that residual malaria transmission may not be solely driven by *An. funestus s.l.,* but also by these ecologically versatile secondary vectors. Greater use of expanded vector surveillance activities, to include 24-h HLC and increased molecular species characterization and comprehensive parasite screening, would help define a more complete picture of malaria transmission.

*Anopheles funestus s.l.* was equally likely to bite indoors or outdoors in the study area. This finding highlights potential limitations of the current core interventions targeting indoor resting and feeding vectors [24]. The use of long lasting insecticidal nets (LLINs), IRS and window screening could be considered for integrated vector control in public settings such as schools where they may contribute to risk reduction for key populations that are difficult to target at home. However, the risk of outdoor and daytime exposure to *An. funestus s.l.* bites in public spaces as well as at home further highlights the operational challenges associated with these core interventions and the need to develop new tools and approaches.

The 24-h surveillance study revealed that while 87.6% of *An. funestus s.l.* bites occurred during the standard night-time surveillance window (18:00–06:00h), 12.4% of bites occurred outside this period, primarily in the late morning (06:00–10:00). This temporal extension indicates that *An. funestus s.l.* is not strictly nocturnal, as often assumed, but exhibits substantial daytime activity in the study area of Western Zambia. This opportunistic behaviour of *An. funestus s.l.* presents a critical gap in existing malaria control strategies.

Late morning biting by *An. funestus s.l.* at schools has also been reported in other countries [12, 13], and is especially concerning considering that asymptomatic school children who are at high risk of exposure to *An. funestus* bites during the school day represent key drivers of transmission in high endemicity settings [25]. In this study, mosquito biting activity at school sites exhibited a delayed onset (22:00–23:00 outdoors and 23:00–24:00 indoors), with significant indoor activity persisting until 10:00.

This pattern underscores potential exposure to vector biting in students and staff during the late morning hours, as they begin their daily activities. Similarly, mosquito biting in markets began around midnight, peaking between 04:00 and 06:00, aligning with human activity in high-traffic areas such as bus stations, nightclubs, bars, restaurants, shops, and markets.

The variability observed in temporal biting patterns and locations underscores the opportunistic behaviour of *An. funestus s.l.* in Western Zambia, enabling it to exploit a variety of human activities and habitats. This could contribute to the high levels of malaria transmission that persist in Western Zambia despite high coverage of core vector control interventions and should be investigated further.

While this study provides valuable insights into mosquito biting patterns, certain limitations should be acknowledged to contextualize the findings. The analysis relied on calculating the average number of bites per person-hour independently for each location and then aggregating these rates by location-hour to estimate an overall daily risk. However, sampling efforts were imbalanced across the collection venues, with limited collections conducted in public spaces. This uneven distribution may not be representative and may have resulted in over- or under-estimations of *An. funestus s.l.* biting rates in these areas. Furthermore, the location of the clusters included in this pilot study may have introduced some location bias in the public space sampling frame, as the two communities with school-based collections were both located in Kaoma district. Therefore, direct comparisons of the number of bites per person-hour across locations should be made with caution and future studies should aim for more balanced sampling approaches, including increasing the frequency of collections in public spaces.

Additionally, the estimated biting rates were based solely on structured human landing catch (HLC) data and do not account for variations in human behaviour. For instance, while there may be a theoretical risk of exposure to *An. funestus s.l.* bites inside a school building at 01:00 h, it is unlikely that individuals would be present and sleeping at this location at that time in Western, Zambia. Future vector surveillance efforts should simultaneously collect data on corresponding human activities and calculate behaviour-adjusted biting risk as outlined by [5, 26–28]. By addressing these limitations, future work can refine our understanding of mosquito-biting patterns and improve the robustness of exposure risk assessments.

## Conclusion

These results document significant exposure to *An. funestus* bites indoors and outdoors, in public spaces such as schools and markets, and late in the morning until 11:00 h in Western Province, Zambia. The flexible, adaptable blood-feeding behaviours exhibited by the dominant malaria vector in Western Province highlight important gaps in protection offered by current vector control strategies, underscoring the need to expand vector surveillance efforts to better characterize the full scope of transmission risk and to develop tailored integrated vector control strategies to target transmission occurring outdoors in public places during daytime.

## Supplementary Information

**Additional file 1.**

## Data Availability

The datasets used and/or analysed during the current study are available from the corresponding author on reasonable request.

## Abbreviations

An.: Anopheles
ATSB: Attractive targeted sugar bait
b/d: Bites per day
CI: Confidence interval
cRCT: Cluster-randomized controlled trial
DEBS: District Education Board Secretary
DHO: District Health Office
EIR: Entomological inoculation rate
GLM: Generalized linear model
HLC: Human landing catch
HH: Household
h: Hours
IRS: Indoor residual spraying
ITN: Insecticide-treated net
IVCC: Innovative vector control consortium
LLINs: Long-lasting insecticidal nets
MoH: Ministry of Health
NMEC: National Malaria Elimination Centre
PATH: Program for Appropriate Technology in Health
PHO: Provincial Health Office
RDT: Rapid diagnostic test
RR: Rate ratio
SDC: Swiss Agency for Development and Cooperation
SE: Standard error
s.l.: Sensu lato
s.s.: Sensu stricto
SR: Sporozoite rate
WHO: World Health Organization
