# Supplementary material for: Daytime and public space exposure to Anopheles funestus bites in Western Province, Zambia: implications for malaria surveillance and control
Source: Malar J. 2025 Apr 18;24:128. doi: 10.1186/s12936-025-05363-0 (PMC12008870; doi:10.1186/s12936-025-05363-0)
Supplement: Supplementary file 1 — Additional file 1. [file 12936_2025_5363_MOESM1_ESM.docx]

Table S1. Average number of bites per day recorded during the 24-hour human landing capture collections, by mosquito species.

|  | Household | |  | Market |  |  | School |  |  | Aggregate |  |  |
| --- | --- | --- | --- | --- | --- | --- | --- | --- | --- | --- | --- | --- |
|  | 9,600 collection hours | |  | 192 collection hours | |  | 192 collection hours | |  |  | |  |
|  | Indoor | Outdoor | **Total** | Indoor | Outdoor | **Total** | Indoor | Outdoor | **Total** | Indoor | Outdoor | **Total** |
| *An. funestus s.l.* | 1.02 | 1.25 | **2.26** | 1.00 | 1.25 | **2.25** | 1.50 | 0.88 | **2.38** | 3.52 | 3.37 | **6.89** |
| *An. squamosus* | 0.52 | 0.69 | **1.21** | 0.25 | 0.00 | **0.25** | 0.00 | 0.13 | **0.13** | 0.77 | 0.82 | **1.58** |
| *An. tchekedii* | 0.59 | 0.58 | **1.17** | 0.38 | 0.88 | **1.25** | 0.00 | 0.00 | **0.00** | 0.96 | 1.46 | **2.42** |
| *An. tenebrosus* | 0.24 | 0.21 | **0.44** | 0.38 | 0.50 | **0.88** | 0.00 | 0.13 | **0.13** | 0.61 | 0.83 | **1.44** |
| *An. gambiae s.l.* | 0.11 | 0.27 | **0.38** | 0.13 | 0.13 | **0.25** | 0.00 | 0.25 | **0.25** | 0.24 | 0.65 | **0.88** |
| *An. coustani* | 0.06 | 0.06 | **0.12** | 0.00 | 0.00 | **0.00** | 0.00 | 0.00 | **0.00** | 0.06 | 0.06 | **0.12** |
| *An. gibinsi* | 0.01 | 0.01 | **0.02** | 0.00 | 0.00 | **0.00** | 0.00 | 0.00 | **0.00** | 0.01 | 0.01 | **0.02** |
| *An. brunipes* | 0.00 | 0.00 | **0.00** | 0.00 | 0.00 | **0.00** | 0.00 | 0.13 | **0.13** | 0.00 | 0.13 | **0.13** |
| *An. maculipalpis* | 0.00 | 0.00 | **0.01** | 0.00 | 0.00 | **0.00** | 0.00 | 0.00 | **0.00** | 0.00 | 0.00 | **0.01** |
| *An. flavicosta* | 0.00 | 0.00 | **0.00** | 0.00 | 0.00 | **0.00** | 0.00 | 0.00 | **0.00** | 0.00 | 0.00 | **0.00** |
| *An. pharoensis* | 0.00 | 0.00 | **0.00** | 0.00 | 0.00 | **0.00** | 0.00 | 0.00 | **0.00** | 0.00 | 0.00 | **0.00** |
| Total | 2.54 | 3.07 | **5.61** | 2.13 | 2.75 | **4.88** | 1.50 | 1.50 | **3.00** | 6.16 | 7.32 | **13.48** |

|  |  | **Household** | | **School** | | **Market** | |  |  |  |  |
| --- | --- | --- | --- | --- | --- | --- | --- | --- | --- | --- | --- |
|  | **Hour** | **Indoor** | **Outdoor** | **Indoor** | **Outdoor** | **Indoor** | **Outdoor** | **Total Bites** | **(%)** | **Total Bites** | **(%)** |
|  | **12:00** | 0.000 | 0.000 | 0.000 | 0.000 | 0.000 | 0.000 | 0.00 | 0.0% | 0.02 | 0.3% |
|  | **13:00** | 0.000 | 0.000 | 0.000 | 0.000 | 0.000 | 0.000 | 0.00 | 0.0% |  |  |
|  | **14:00** | 0.000 | 0.000 | 0.000 | 0.000 | 0.000 | 0.000 | 0.00 | 0.0% |  |  |
|  | **15:00** | 0.010 | 0.003 | 0.000 | 0.000 | 0.000 | 0.000 | 0.01 | 0.2% |  |  |
|  | **16:00** | 0.000 | 0.000 | 0.000 | 0.000 | 0.000 | 0.000 | 0.00 | 0.0% |  |  |
|  | **17:00** | 0.005 | 0.005 | 0.000 | 0.000 | 0.000 | 0.000 | 0.01 | 0.1% |  |  |
| Standard HLC Surveillance Window | **18:00** | 0.008 | 0.015 | 0.000 | 0.000 | 0.000 | 0.000 | 0.02 | 0.3% | 6.03 | 87.6% |
|  | **19:00** | 0.010 | 0.020 | 0.000 | 0.000 | 0.000 | 0.000 | 0.03 | 0.4% |  |  |
|  | **20:00** | 0.020 | 0.033 | 0.000 | 0.000 | 0.000 | 0.000 | 0.05 | 0.8% |  |  |
|  | **21:00** | 0.043 | 0.045 | 0.000 | 0.000 | 0.000 | 0.000 | 0.09 | 1.3% |  |  |
|  | **22:00** | 0.060 | 0.078 | 0.000 | 0.125 | 0.000 | 0.000 | 0.26 | 3.8% |  |  |
|  | **23:00** | 0.128 | 0.138 | 0.125 | 0.000 | 0.000 | 0.000 | 0.39 | 5.7% |  |  |
|  | **0:00** | 0.110 | 0.150 | 0.000 | 0.000 | 0.125 | 0.250 | 0.64 | 9.2% |  |  |
|  | **1:00** | 0.103 | 0.153 | 0.250 | 0.500 | 0.000 | 0.250 | 1.26 | 18.2% |  |  |
|  | **2:00** | 0.120 | 0.193 | 0.500 | 0.000 | 0.375 | 0.000 | 1.19 | 17.2% |  |  |
|  | **3:00** | 0.145 | 0.135 | 0.125 | 0.000 | 0.000 | 0.125 | 0.53 | 7.7% |  |  |
|  | **4:00** | 0.123 | 0.133 | 0.000 | 0.125 | 0.000 | 0.375 | 0.76 | 11.0% |  |  |
|  | **5:00** | 0.090 | 0.110 | 0.000 | 0.000 | 0.375 | 0.250 | 0.83 | 12.0% |  |  |
|  | **6:00** | 0.028 | 0.020 | 0.125 | 0.000 | 0.000 | 0.000 | 0.17 | 2.5% | 0.83 | 12.1% |
|  | **7:00** | 0.015 | 0.013 | 0.000 | 0.125 | 0.125 | 0.000 | 0.28 | 4.0% |  |  |
|  | **8:00** | 0.000 | 0.003 | 0.125 | 0.000 | 0.000 | 0.000 | 0.13 | 1.8% |  |  |
|  | **9:00** | 0.000 | 0.000 | 0.125 | 0.000 | 0.000 | 0.000 | 0.13 | 1.8% |  |  |
|  | **10:00** | 0.003 | 0.000 | 0.125 | 0.000 | 0.000 | 0.000 | 0.13 | 1.8% |  |  |
|  | **11:00** | 0.000 | 0.003 | 0.000 | 0.000 | 0.000 | 0.000 | 0.00 | 0.0% |  |  |
|  | **Total Bites** | 1.02 | 1.25 | 1.50 | 0.88 | 1.00 | 1.25 | **6.89** |  | **6.89** | **100%** |
|  | **(%)** | 14.8% | 18.2% | 21.7% | 12.7% | 14.5% | 18.1% | **100%** |  |  |  |
|  | **Total Bites** | 2.26 | | 2.38 | | 2.25 | | **6.89** |  |  |  |
|  | **(%)** | 32.9% | | 34.4% | | 32.6% | | **100.0%** |  |  |  |
|  |  |  |  |  |  |  |  |  |  |  |  |

Figure S1. *Anopheles funestus* s.l biting rate heat map showing comparative biting rates by time and location.
